# The American Ophthalmological Society

**Published:** 1888-08

**Authors:** 


### AMERICAN OPHTHALMOLOGICAL SOCIETY.

The twenty-fourth annual meeting was held at New London, Connecticut, July 18 and 19, 1888.

The President, Dr. W. F. Norris, in the chair.

A memorial of Dr. C. R. Agnew was read by Dr. H. D. Noyes. Committees were appointed to draw up resolutions concerning the death of Dr. E. G. Loring and that of Dr. Joseph Aub.

A Contribution to the Treatment of Membranous Opacities of the Vitreous.—Dr. Charles S. Bull, of New York, read a paper which contained histories of fifteen cases of membranous opacities of the vitreous in which he had resorted to the operation first performed by Von Graefe, an account of which was published in 1863. Internal treatment and local applications had no effect on those opacities of the vitreous demanding this operation. According to the nature of the case, he employed the discission needle, the sharp-pointed cataract knife, or a needle with a broader point.

Dr. F. Buller, of Montreal, gave the history of a case of pulsating exophthalmia cured by ligation of the common carotid.

Dr. Swan M. Burnett, of Washington, read a paper giving an analysis of the refraction of 576 healthy human corneæ examined with the ophthalmometer of Joval and Schiotz.

Dr. J. B. Emerson, of New York, read the history of a case of progressive hypermetropic astigmatism.

Dr. E. Jackson, of Philadelphia, reported a case of œdema of the choroid and retina, and also exhibited a new form of cataract knife.

New instruments were presented by Dr. Tansley, consisting of a lachrymal syringe, modified probes for enlarging the lachrymal canal, a clamp to close the entrance to the lacrhymal duct and prevent the passage of atropine solution into the nose and throat, and an improved style.

Aluminium lachrymal probes were shown by Dr. Theobald. Being very smooth and slippery, they give less pain than silver probes, and do not oxidize.

Dr. Risley, Dr. Theobald, and Dr. Noyes gave the results of some examinations as to the size of the lachrymal canal in the dry skull, and the lesson drawn was that, inasmuch as the size of the canal varied much in different subjects, and perhaps also on the two sides in the same subject, one could be governed in the use of probes only by actual trial in each case.

Symptomatic Myopia.—Dr. Mittendorf said that the fact that myopia as a symptom of certain affections of the eye was so little appreciated had led him to prepare this paper. Traumatism might give rise to symptomatic myopia, but the condition occurred more commonly with some disease. In some rare cases it had been caused by dislocation of the lense forward.

Hysterical Blindness in the Male.—Dr. W. O. Moore, of New York, stated in a paper that Briquet had found, out of one thousand cases of hysteria, fifty in males, while other authors had found the proportion of males to females suffering from the disease as one to fifteen. From 1875 to 1885, in France, no fewer than five theses on hysteria in the male had appeared. In males the symptoms lasted longer than in females, and they were more likely to occur in weak and effeminate subjects.

